# Exposure to soil environments during earlier life stages is distinguishable in the gut microbiome of adult mice

**DOI:** 10.1080/19490976.2020.1830699

**Published:** 2020-12-31

**Authors:** Wenjun Liu, Zheng Sun, Chen Ma, Jiachao Zhang, ChenChen Ma, Yinqi Zhao, Hong Wei, Shi Huang, Heping Zhang

**Affiliations:** a Key Laboratory of Dairy Biotechnology and Engineering, Inner Mongolia Agricultural University, Hohhot, China; b Single-Cell Center, Qingdao Institute of Bioenergy and Bioprocess Technology, Chinese Academy of Sciences, Qingdao, Shandong, China; c College of Food Science and Technology, Hainan University, Haikou, Hainan, China; d Department of Preventive Medicine, University of Southern California, Los Angeles, CA, USA; e Key Laboratory of Agricultural Animal Genetics, Breeding, and Reproduction of the Ministry of Education & Key Laboratory of Swine Genetics and Breeding of Ministry of Agriculture and Rural Affairs, Huazhong Agricultural University, Wuhan, P. R. China

**Keywords:** Germ free mice, Gut microbiota, Soil environment exposure, Migrations, Metagenome

## Abstract

Environmental exposure during earlier life stages can govern the assembly and development of gut microbiota, yet it is insufficiently understood. In this study, ex-germ-free mice were cohoused with distinct soil-microbiota (from desert, steppe, and forest) beddings within 60 days after birth and subsequently transferred to new soil beddings from 60 to 90th day. Using metagenomic shotgun sequencing, firstly, we found soil microbes from natural environments (birthplace) greatly influenced the gut community assembly in the housing experiment. About 27% microbial species and 12% functional components that associated with birthplaces at Day 60 were still discriminatory of birthplaces after transferring mice to new environments. Moreover, prior soil-exposure types are associated with the magnitude of temporal microbiome change due to environmental shifts. The appropriate soil-exposure (e.g., steppe) might help mice gut microbiome adapt to changing environments or host development. Our study demonstrated the continuous soil-exposure history earlier is associated with the gut microbiome individuality and development later.

## Introduction

Environmental exposure, especially in the earlier life stages, has a profound influence in human health. It has been suggested that such effect is, at least in part, mediated by shaping the individual gut microbiota, while in turn, microbial colonizers in the gastrointestinal tract affect the host physiology/development in the life span.^1–4^ The advances in the related field could further guide development of new approaches for modulating the risk for ecological invasion by various natural-environment-derived microbes related to human migration, deepen our understanding of how environmental microbial exposures shape the development of our immune systems,^5–7^ and help direct the design of more effective strategies for microbiome modification.

To date, studies aimed at identifying the environmental factors associated with gut microbiota assembly have largely focused on host genetics^8–10^ and diet^8,11,12^ etc. The soil exposure during earlier life stages or the rich microbiota within soil environment exhibited a larger impact on host health than is often assumed.^5,6^ Nonetheless, few studies have elucidated the influence of soil-microbiota exposures on assembly and resilience in gut microbiome in an integrative manner. Firstly, it is unclear how different is the colonization ability of microbes from diverse soil environments. Second, it is likely that host gut niches frequently interact with distinct communities due to soil environmental changes especially in the early stage of life, leading to the divergence of gut microbial communities. However, it is largely unknown if/how these earlier environmental exposures linked to any differences of gut microbiome in response to subsequent environmental disturbance. Third, priority effect^3,13^ has suggested that the timing of bacterial arrivals in the gut has profound influence on shaping the gut microbiota in the early life, however, developmental and evolutional feedback between host and microbiome in the following life events are not often evident in studies.^14^ In addition, previous studies^6,15^ were limited to analyze species-level microbial colonizers due to the resolution afforded by conventional approaches, such as 16S rRNA sequencing.^16^ In contrast, metagenomic whole-genome shotgun sequencing directly sequences the full genetic repertoire in a microbial community enabling a comprehensive and in-depth taxonomic and functional profiling of the community.^17^

In the present study, to assess the colonization and competition potential of components of soil microbiota that reside in distinct habitats, we developed a gnotobiotic mouse model that mimics soil microbiota colonization and development in the mouse gut during emigration-like environmental changes. Totally, 90 germ-free mice were randomized into three groups (30 per group) and each group was raised under an assigned-simulated environment for 60 days, using different natural soil samples collected from steppe, forest or desert habitats. In addition, two-thirds of mice were randomly selected at Day 60 from each group and transferred to new cages with bedding soil from the other two environments, respectively, for 30 more days while the rest 10 mice (as a control group) were housed under the original environment they were initially housed. Stool samples were collected again at Day 90 for further analysis, for the purposes of examining the microbial interaction between soil environment and gut. Our study inquired how soil microbes colonize and develop in the mice gut introduced by environmental exposure rather than well-documented transplantation. This approach can further facilitate identification of environmental-dependent soil-derived colonizers in mammalian host gut that affect any aims related to modulating gut microbiota toward a “health” state.

## Results

### Profound differences in the gut-selected microbiome of germ-free mice under different earlier environmental exposures

**Figure 1.**
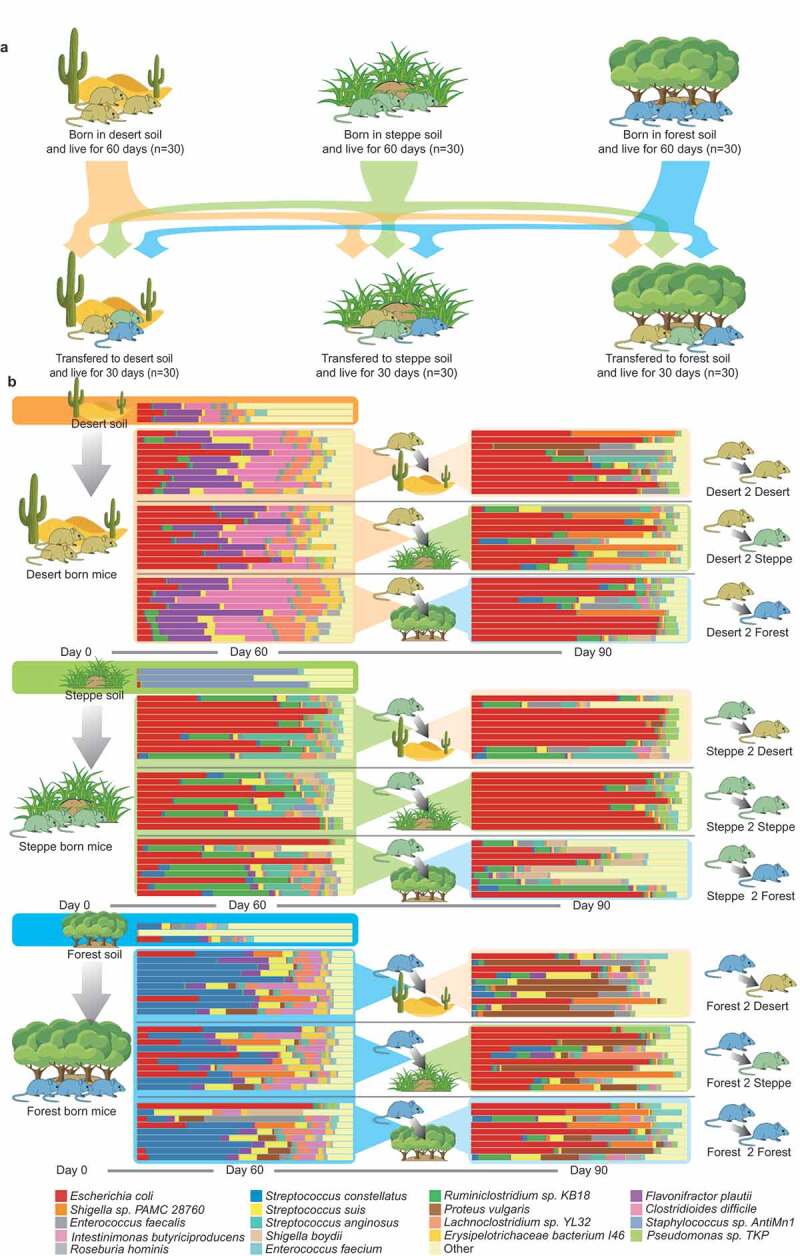
**The taxonomy analysis of fecal microbiomes in this study**. (a) Experimental design. Germ free mice were born in cages with soil from desert, steppe and forest and raised for 60 days. After Day 60, mice under different soil conditions were cross-transferred. For example, 20 mice in the desert group were randomly selected and transferred to other environments (half of them were transferred to steppe soil environment and the rest were transferred to forest soil environment), ten mice were still raised in desert environment, so did mice in the other two groups. Color of mice refers to the birthplace for instance yellow stands for desert-originated mice, green for steppe-originated mice and blue for forest-originated mice. (b) The bar plots indicate the taxonomic compositions of all fecal samples at the species level from this study. Only metagenomic species with more than 0.1% relative abundance within a gut microbiome were presented while the remaining species were together presented as “others” in the bar plot

**Figure 2.**
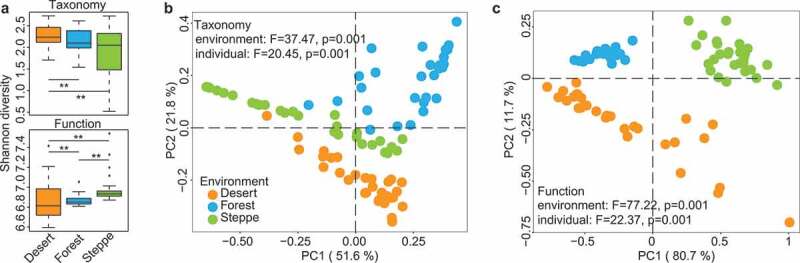
**Microbial diversity analysis of gut microbiome of mice born in different environments**. (a) Alpha diversity of taxonomic composition (at species level, upper panel) and KEGG function (lower panel), both have significant *P* value among the three groups based on Kruskal-Wallis Test. (b-c) Principal coordinates analysis of gut microbiomes (taxonomy and function) in mice from different environments at Day 60, PCoA plot at left panel is based on taxonomic composition at the species level, and the right panel is based on microbial function. The results of PERMANOVA related to confounding factors such as groups (environments) and host IDs (individuals) were also marked on the plots. Even though host individuality did influence both taxonomy and function of gut microbiome, host groups related to soil environments that mice was born has a greater effect size than host IDs

**Figure 3.**
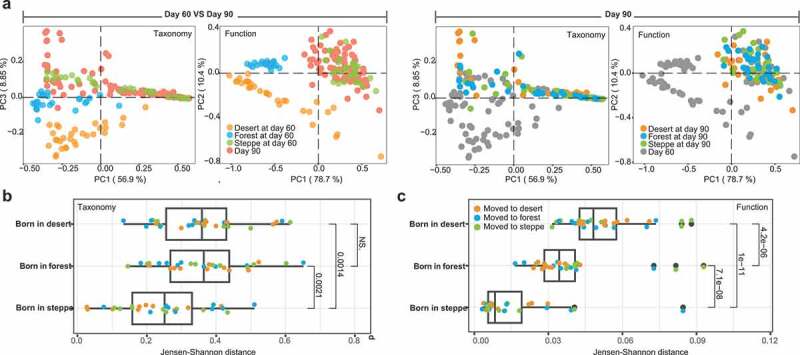
**The alteration in the gut microbiome associated with cross-environment migration**. (a) Principal Coordinates Analysis of taxonomic and functional profiles in the mice gut microbiome before (Day 60) and after (Day 90) environment transferring. The left panel shows the different of taxon and function between Day 60 (mice from the three groups were colored with orange, blue and green for desert, forest and steppe as their birthplaces) and Day 90, mice at Day 90 were all colored with red to different with Day 60. On contrary, in the right panel the Day 60 mice were colored with gray to different with Day 90. (b) Taxonomic change based on Jensen-Shannon distance metric from Day 60 to Day 90. Mice were grouped by their birthplaces to illustrate its influence on immigration, points were colored by the new environments that mice co-housed with at Day 90. (c) Functional change in the gut microbiome based on JSD metric from Day 60 to Day 90, which was mainly grouped by birth places. Points were colored by the new environments that mice co-housed with at Day 90

We introduced soil microbiota from different habitats (steppe, forest, and dessert) to separate groups of germ-free (GF) mice (Figure 1a**; Methods**) right after birth. Newly born GF mice were raised in the simulated environments (steppe, forest, and dessert) for 60 days. Soil samples used for cage bedding were collected from Gele Mountain Forestry Park, Chongqing (106.45° E/29.53° N, forest), Xilin Gol Grassland Nature Reserve, Inner Mongolia (113.83° E/42.23° N, steppe) and Kubuqi Desert Park, Inner Mongolia (109.79° E/39.62° N, desert, **Methods**). We first assessed and compared the colonization ability of components of microbiota that reside in distinct birthplaces (habitats). Shotgun metagenomics sequencing technique was applied to explore the functional and taxonomic differences in mouse gut microbiome between the three groups (Figure 1b**, Methods**): (1) Microbial alpha diversity analysis (i.e. Shannon diversity) revealed significant functional and taxonomic differences in gut-selected microbiome among three groups, (Figure 2a, Kruskal-Wallis Test, Taxonomy: *p* = .023 Function: *p* = .001); (2) Principal coordinates analysis (PCoA) based on the Jensen-Shannon distance metric primarily clustered gut microbiomes from three groups of mice both taxonomically and functionally (Figure 2b, 2c); (3) PERMANOVA results indicated the birthplace/living-environment derived clustering based on gut functional diversity is more distinct than that based on taxonomical diversity, even though they were both strongly influenced by living condition. Therefore, we reasoned that birthplace profoundly shaped the compositions and metabolic functions of gut microbial communities in mice during the early stages of life.

To elucidate what exogenous microbiota members were selected by mice gut when continuously exposed to distinct environments (soil) during earlier life stages, we compared the difference in the diversity of soil microbiota and gut-selected microbial communities in gnotobiotic mice at Day 60 (Figure 1b). *First*, the gut communities selected from desert and forest soil environments maintained a greater proportion of microbial richness relative to environmental “input” microbiota than did those from the steppe environmental microbiota. In the dessert soil, a total of 352 microbial species (mean Shannon index: 3.23) were identified while 251 species (71.3% relative abundance) out of them further established in the mice gut and accounted for 99.2% abundance of host gut microbiotas (mean Shannon index: 2.25) averagely. Likewise, 211 (76.2%) out of 290 species (mean Shannon index: 2.63) in forest soil were colonized in the mice gut accounting for on average 95.4% abundance of gut-selected microbiota (mean Shannon index: 2.11). In contrast, among 443 species (mean Shannon index: 1.36) identified in steppe, 209 species (47.2%) established and accounted for on average 98.3% abundance of gut microbiota (mean Shannon index: 1.81) of steppe born mice. *Secondly*, we further found gut microbial communities selected from desert and forest soil maintained a smaller beta diversity (Jensen-Shannon distance = 0.19 and 0.34) of taxonomic profiles relative to their “input” community than did communities from steppe (Jensen-Shannon distance = 0.60) soil environments.

### Taxonomic and functional indicators in the gut microbiota of prior exposure to different environments

We further identified environment indicative microbial colonizers in the earlier life (i.e., Day 60 after birth, **Figure S2a S2b** left panel). Firstly, we identified birthplace-specific microbes in the mice gut. Mice in steppe group contain 19 unique species accounting for 0.03% relative abundance in steppe mice gut microbiota (unique properties were not shown in the figure); forest mice contain 29 unique species (relative abundance is 0.07%) and dessert mice contain 57 (relative abundance is 0.13%). Secondly, among the remaining 140 shared species, we found 37 species were differentially abundant among the three groups. 16 species belonging to Firmicutes, Proteobacteria, and Spirochetes were enriched in steppe-born mice compared to the other two groups. Seven species from Firmicutes, and two species from Actinobacteria had higher abundances in desert born mice compared with other two groups. The remaining 11 taxa showed significantly more abundant in forest born mice.

Next, we characterized the functional properties of the mice gut-selected microbiota by examining differentially abundant microbial genes and enriched metabolic pathways among desert-, forest- and steppe-born mice on Day 60 (**Methods**). Shotgun Illumina reads generated from fecal community DNA were used to query the Kyoto Encyclopedia of Genes and Genomes for KEGG Orthology group (KO). Reads collected from all gut-selected microbiomes were assigned to a total of 4011 KOs, among which 3883 KOs were shared among different environments, and 3893 KOs were differentially abundant among the three groups. In the following KEGG pathway enrichment analysis, we identified 31 significantly enriched metabolic pathways in successful colonizers among distinct soil environments, which were dominantly enriched in steppe-born mice gut (**Figure S3**), involving bacterial chemotaxis (ko02030) and flagellar assembly (ko02040), two-component system (ko02020) etc., suggesting that the microbial metabolic functions are likely correlated with environmental adaptation. Remarkably, active hydrocarbon metabolic processes in gut microbiota in the steppe group mice can be evident by the presence of enriched pathways involved in amino sugar and nucleotide sugar metabolism (ko00520), butanoate metabolism (ko00650), citrate cycle (TCA cycle) (ko00020), fructose and mannose metabolism (ko00051), glycolysis and Gluconeogenesis (ko00010), pentose, and glucuronate interconversions (ko00040), propanoate metabolism (ko00640), pyruvate metabolism (ko00620), starch, and sucrose metabolism (ko00500).

### The cross-environmental exposures of mice from day 60 to 90

In the following experiment, we tested the capacities of taxa comprising birthplace-derived communities to compete for colonization of the mouse gut during cross-environment migration. After 60-day establishment of environmental microbes in the mice gut, two thirds of mice within desert, forest or steppe group (N = 60) were transferred to a different soil environment and housed for another 30 days, while one third of mice within each group (N = 30) stayed in the same environment for 30 days (as control). This experimental design allowed us to determine (1) whether/how migration can reshape indigenous gut microbiota established by birthplace-derived soil microbes; (2) whether/which organisms from the new environmental communities were capable of invading the established gut communities, while controlling temporal variations (such as age) in the gut microbiota development.

### The birthplaces-dependent magnitude of changes in gut microbiomes during the cross-environmental migration

After transferring mice to a new environment, gut microbiome in all groups were significantly altered in both functional and taxonomic compositions from Day 60 to Day 90 as observed in the PCoA (Figure 3a left panel; Day 60 VS 90, taxonomy: F = 30.69, *p* = .001; function: F = 21.70, *p* = .001, PERMANOVA). Furthermore, birthplaces can determine the magnitude of the alterations in mice gut microbiome due to environmental transferring. We compared the taxonomic and functional profiles based on the Jensen-Shannon distance metric between host groups related to the soil microbiome types and before and after transferring. (Figure 3b). Based on the species-level taxonomic profiles, the migration-derived change in steppe-originated mice gut microbiome is significantly less than forest-originated (*p* = .0014, Wilcoxon rank-sum test) or dessert-originated mice (*p* = .0021, Wilcoxon rank-sum test). Moreover, from overall functional profiles of successful colonizers, we observed more pronounced birthplace-dependent differences related to environmental shifts. The least magnitude of change before and after shifts in the co-housing soil types was identified in the steppe-originated gut microbiome of mice (Figure 3c), indicating that the steppe-originated soil microbiome might establish and stabilize earlier in the gut, while highly resistant to other types of soil microbiota in the new environment. Interestingly, we next found that gut microbial communities originated from different soil types consistently converged toward the steppe-associated gut communities. The reproducible nature of these changes in all isolators suggests that non-stochastic, selective forces  that were potentially driven by the compatibility of the gut microbiome with host factors played an important role in shaping these communities.

### Both microbial taxonomic and functional signatures associated with birthplaces persisted after cross-birthplaces migrations

Given a strong impact of soil exposure on gut microbiota during earlier life stages, and even post-migration phase, we further explored both microbial taxonomic and functional markers reflecting such birthplace-dependent differences. We performed the differentially abundant analysis, respectively, on microbiome data grouped by birthplace-environment types on Day 60, new environment types on Day 90, and birthplace environment types on Day 90 and further compared the microbial associations to original and new environmental types on Day 90 relative to those associating birthplace-environment types on Day 60. Further, a microbial birthplace-associated marker was defined as a microbe/functional gene significantly associated with environmental types at both Day 60 and 90 and with consistent directionality of enrichment.

**Figure 4.**
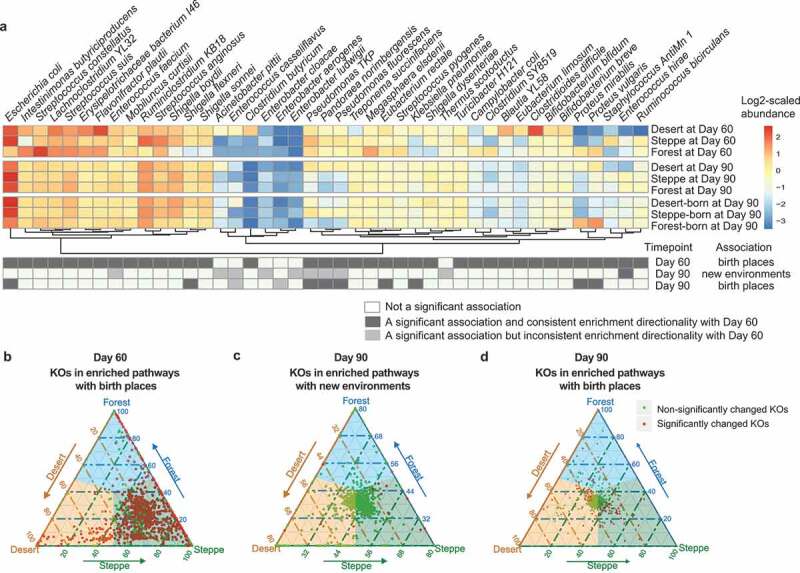
**The soil exposure from the earlier life stages can be distinguishable in the post-transferring gut microbiomes**. (a) The heatmap indicates the environment-associated taxonomic markers at Day 60, Day 90 grouped by current environment, and Day 90 grouped by birthplaces. Differentially abundant species at Day 60 were marked as black cells in the first row, then differentially abundant species at Day 90 grouped by post-transferring environments and birthplaces were also marked as black cells in the second and third row, while those differentially abundant species but having inconsistent direction of the enrichment at Day 60 were colored by gray. (b) Abundance comparison of KOs in related pathways significantly enriched among birthplaces at Day 60. Light green points refer to those KOs who were not significantly changed among three groups and red for those significantly changed KOs. The closer the point to the apex of triangle the higher abundance of this KO in the corresponding group. (c) Abundance comparison of KOs in related pathways significantly enriched among new environments at Day 90. (d) Abundance comparison of KOs in related pathways significantly enriched among birthplaces at Day 90 grouped by birth place

We first performed such association analysis on the taxonomic profiling data. On Day 90, totally 10 species (Figure 4a**, Figure S2a**, right panel) were differentially abundant among the new living environments, while 11 species (**Figure S2b**, right panel) were still indicative of the birthplaces after cross-birthplaces migration. Firstly, among the 10 new-environment-associated markers, three species from *Enterococcus* (*Enterococcus casseliflavus, Enterococcus hirae* and *Enterococcus faecium*) and one species from *Thermus* (*Thermus scotoductus*) were found enriched in forest mice (at Day 90). Two species of *Enterobacter* (*Enterobacter cloacae* and *Enterobacter ludwigii*) were found more abundant in steppe mice and the rest four (*Acinetobacter pittii, Pandoraea norimbergensis, Pseudomonas fluorescens* and *Pseudomonas TKP*) were found enriched in desert mice. Interestingly, only one species-level marker had the same change tendency compared to Day 60, while totally five species were differentially abundant among environmental types on both Day 60 and 90 (Figure 4a). This suggested that the cross-environment migration did alter gut microbial communities, but gut bacteria associated with soil types in new cohousing environments were far less than and different from those found prior to migratione.

On the contrast, we found more taxonomic markers (N = 9) still associated with birthplace on Day 90 after cross-environmental migration, accounting for 27% of all birthplaces indicative species previously identified on Day 60. Among these nine birthplace-discriminatory taxonomic markers at Day 90, five species (*Escherichia coli, Pandoraea norimbergensis, Pseudomonas fluorescens, Pseudomonas TKP* and *Shigella flexneri*) were enriched in steppe born mice, two species (*Eubacterium rectale* and *Klebsiella pneumoniae*) were more abundant in desert born mice and the rest two species (*Proteus mirabilis* and *Proteus vulgaris*) were enriched in forest born mice. Other than these nine metagenomic species associated with birth places at Day 90, only two extremely low abundant species (*Enterobacter aerogenes* and *Enterococcus casseliflavus*) had the inconsistent enrichment pattern with that identified at Day 60. Thus, even after environmental changes, the microbial signature was still discriminative of birthplaces in the gut of adult mice.

Next, we examined if functional signatures in the gut microbiome associated with birthplace at Day 60 would be still discriminative at Day 90. We further analyzed the differentially abundant functional genes and enriched pathways on Day 90's data grouped by both new environmental types and birthplaces. Firstly, a total of 27 metabolic pathways (Figure 4b, right panel; **Figure S4**) were significantly associated with new environmental types based on 379 differentially abundant functional genes (KOs). Interestingly, the top 20 enriched pathways (according to a reporter Z score at the pathway level, see **Methods**) at Day 90 associated with new environments were highly consistent with those associated with birthplaces at Day 60 (Figure 4b, right panel). Furthermore, among those 379 KO markers, 134 had the consistent direction of associations with those associated with birthplaces at Day 60. Given two thirds of mice in each new environment were transferred from different soil environments, the large number of consistent functional associations between Day 60 and 90 are noticeable. These may suggest that functional capacity in the gut of migrants had well adapted to new environments. Among these 27 enriched pathways indicative of new environments, eight related to carbohydrate metabolism (ko00650, Butanoate metabolism; ko00020, Citrate cycle; ko00051, Fructose and mannose metabolism; ko00052, Galactose metabolism; ko00010, Glycolysis/Gluconeogenesis; ko00040, Pentose and glucuronate interconversions; ko00640, Propanoate metabolism; ko00620, Pyruvate metabolism) and five pathways related to environmental adaption (ko02030, Bacterial chemotaxis; ko02040, Flagellar assembly; ko03070, Bacterial secretion system; ko02060, Phosphotransferase system; ko02020, Two-component system) and other pathways are mainly related to nutrition metabolism.

Next, we explored birthplace-discriminatory functional components at Day 90 by associating microbiome profiles at Day 90 with birthplace environments. This result may indicate the functional capacity of the gut ecosystem to withstand environmental perturbances related to shifts in soil environmental types (i.e., resistance) in the co-housing experiment. Totally, 195 (12%) out of 1567 differentially abundant KOs among birthplaces’ environmental types on Day 90 had the identical directionalities of associations with those at Day 60, which involved in the 31 significantly enriched pathways. It suggested that approximately 12% of microbial functional signature of soil exposure during earlier life stages can be distinguishable in the extraordinarily long term even though the more environmental soil exposure later had greatly disturbed the functional profiles in the established gut microbiome.

## Discussion

Our study showed a clear yet soil-type-dependent effect of soil exposure during earlier life stages on the gut microbiota assembly and its development in response to a new soil environment. By housing experiment, we found extensive soil-dwelling microbiota from a variety of environmental types were able to successfully colonize on mouse gut. In addition, the earlier microbial colonizers can significantly impact the gut microbiome development during the abrupt environmental disturbances that are also suggested by “priority effect”. As a result, a set of microbial taxa/functions in the mouse gut can be persistently discriminative of birthplaces in the post-migration microbiomes (Phase II). However, the taxonomic and functional profiles developed over time (or with host selection) toward a consistent microbiome state in the adult mice in a soil-type-dependent pace. The gut-selected microbiomes from steppe soils may develop fastest to that microbiome state. Hence, our study found that the “priority effect” may only partially explain the impact of early soil exposure on the plasticity of gut microbiota in the later lifespan, while underscoring the role of soil-derived colonization history on the “normal” development of gut microbiome.

The soil-derived microbes colonizing the mammalian gut in the earlier life stages have a long-lasting impact on their gut microbiome, yet such impact was usually overlooked in the previous studies. To date, a large proportion of variations in the human gut microbiota still remains elusive. Studies have shown that an individual’s genetics, diet, environment, lifestyle, and physiological state can make limited contributions (less than 30%) to the variation of the gut microbiome among individuals.^18^ It suggested that other unknown factors (such as underexplored environmental factors) likely shape these microbial communities as well. On the other hand, the environmental samples are often not sufficiently collected or omitted in most of microbiome-focused studies on human/mouse hosts. However, studied hosts were often exposed to and constantly interacting with microbes residing surrounding environments, such as soil microbes.^15^ For example, it is difficult to remove soil microbes from each food, while the soil-derived microbiota likely attached to foods can be inevitably passed through our bodies while nutritional ingredients and components are digested. It is unclear how much of such inoculum successfully passes through our gastric barrier, reaches our intestine and colon, resulting in colonization and whether/how those soil microbial colonizers from different sources/geographic locations can diversify the gut microbiome among individuals. Therefore, until now, scientists still lacked sufficient data to explore soil-derived variations in gut microbiota. We here developed the ex-germ-free mouse model to understand the underexplored effects of co-housing mice with soils during earlier life stages on the later gut microbiome development. We acknowledge this study has potential limitations in the study design or experimental operations, which should be informed for data interpretation. (1) We continuously exposed mice to input soil community from birth to Day 60. Thus, we reported observations about the soil exposures during earlier life stages rather than the short-term exposure in the early life (at birth) that often studied. (2) Furthermore, to avoid the fecal accumulation in living environments, we replaced the soil-bedding with fresh soils every 2 weeks. Despite the fresh soil from a given environmental type can be highly homogeneous, mice could be potentially recolonized by the fresh soil microbiota every 2 weeks until Day 60. Nonetheless, we still believe that it has particularly important values to the community. We found that most of those alien microbes in diverse soil environments are capable of colonizing the mouse gut persistently. Furthermore, these earlier microbial colonizers exhibited the profound effects on the gut microbiome in response to environmental disturbance in the later life.

The next interesting question is whether a particular source of soil microbes can be the most “compatible” to host gut development. We speculated that steppe can be a superior source of microbial colonizers that potentially promoted the gut microbiome maturity in mouse gut based on the following observations.

(1) As early as Day 60, we have observed outstanding gut microbiome features enriched in steppe-born mice under the host selection. In Phase I, the steppe-born gut microbiotas have rapidly developed a substantial number of essential functional genes/pathways compared to those in other groups, which primarily modulated carbohydrate metabolisms (such as, amino sugar and nucleotide sugar metabolism, butanoate metabolism, TCA cycle, fructose and mannose metabolism,glycolysis and gluconeogenesis, pentose, and glucuronate interconversions, propanoate metabolism, pyruvate metabolism,starch, and sucrose metabolism) and environmental adaption (such as bacterial chemotaxis, flagellar assembly, two-component system). Furthermore, on Day 60, we found that the gut microbiota of steppe-born mice has the least overlap of microbes with input soil microbiota, suggesting a more rapid developmental rate in the steppe-born mice gut microbiota.

(2) In the environmental transferring phase (Phase II), we tested if the microbiome convergence toward a steppe-born state took place in the control mice (one-third of the mice in each group (n = 10)) who stayed in the same environment for 30 days. For mice in the control groups born from forests or desert who never contacted steppe soil earlier, the taxonomic and functional profile of gut microbial communities still developed toward a steppe-group-associated microbiota, suggesting an unknown yet intriguing developmental effect in the mouse gut microbiota (Figure 3). Likewise, we observed such a developmental pattern for mice that experienced environmental transferring (Figure 3). Notably, despite the gut microbiome convergence along host development, we can still distinguish the soil exposure histories of mice from the gut microbiome in the later time point (Day 90; adulthood) (Figure 4).

(3) The gut-selected microbiota from steppe soils may be more adaptive to host selection or development. Multiple recent studies showed that being exposed humans to environments rich in soil microbes should offset the detrimental changes in the gut microbiota that profoundly affected human innate immunity due to urbanization.^5,13^ This suggested, if human ancestors long-lived in soil-rich environments, then earlier soil exposure history might help us understand if we can or how to modulate the gut microbiota of descenders for digestive health by designing appropriate environmental exposures in the life span. This might hold true for mice. Coincidentally, the ancestral homeland of the house mouse (*Mus musculus*) is likely located in steppe-like environments such as Pothohar Plateau in Pakistan (Auffray et al., 1990). We reasoned that host selection may facilitate the colonization of microbial species derived from steppe soil and enhance their resistance to later environmental selection pressures. Our results provided the clues to find such an appropriate earlier environmental exposure matched with ancestors’ life history, which mice may favor or benefit from in the later host development. It might explain why gut microbiomes consistently converged toward those derived from steppe soils in different host groups. Especially when we extended the gut microbiome surveys to a particularly long period of life span (from Day 0 to 90), we still observed such a strong impact of host selection on the resident gut microbiome that was often omitted. Therefore, steppe-derived microbial exposure may be superior to others in colonizing and developing with mouse gut in the later life.

Contamination is one of the major challenges for studies using a germ-free animal model, thus high operation standards, experimental skills, and decontamination experience are required to avoid such issues. To avoid any cross-group contamination of soil-derived microbes in the bedding, cages from different groups were maintained in independent aseptic isolators (in different rooms). To avoid diet contamination, the food and water fed to mice were strictly sterilized by autoclaving and radiation (with Cobalt-60) during the whole experiment (Methods). Any possible contamination can be evident in gut microbiota of control group where mice were not transferred to any environments. We carefully examined the microbial sources of the gut microbiome for the control mice at Day 90 from the earlier samples. The day-60 gut microbiota of control mice can account for over 99.9% microbial abundance in corresponding mouse gut microbiome at Day 90 (Figure S5), while the relative abundances of individual species can vary between samples from different time points or groups. It strongly suggests that the gut microbiome convergence toward steppe is not introduced by any contamination. However, we also acknowledge that observational results regarding the microbiome convergence might have alterative explanations and warrants further in-depth investigations providing a holistic view of the mouse development.

Collectively, we provided the clear evidence that soil living environment alone can have a long-term effect on the gut microbial structure and functions. We demonstrated that environment–gut interactions during the earlier life stages can explain the gut microbiome individuality in the following life. The strong developmental effect on gut microbiome probably derived from host was also noted in a transition between adolescent to adult mice. Intriguingly, the gut-microbiome developmental rate can strongly associate with earlier environmental exposure to different soil types. Thus, further investigations are needed to thoroughly understand the potential compatibilities of environmental microbiomes, resident gut microbiome and host factors that can be reflected in the overall host development.

## Materials and methods

### Experimental design

In our study, to understand soil environmental factors that operate to allow microbes to colonize the mammalian gut, the soil samples were collected from three different environments in China including Gele Mountain Forestry Park, Chongqing (106.45° E/29.53° N), Xilin Gol Grassland Nature Reserve, Inner Mongolia (113.83° E/42.23° N) and Kubuqi Desert Park, Inner Mongolia (109.79° E/39.62° N) to simulate the three typical ecosystems of forest, steppe and desert. In the initial phase (Phase I), a total of 15 germ-free pregnant mice (BALBc, one mouse per cage) were randomized into three groups and each group was raised under an assigned soil environment before giving birth. Neonatal mice were born and breastfed in soil environments for the first 20 days. After weaning (on Day 20), mice were re-allocated into sex-dependent cages (four cages for each group/environment, 7 or 8 mice in one cage) and raised in the consistent environments until Day 60. To avoid any cross-groups contamination of soil microbes, mice cages from different groups were maintained in three independent aseptic isolators. In the “migration” phase (Phase II), 20 of 30 mice were selected randomly at Day 60 in each group and distributed to the other two environments for 30 more days while the rest 10 mice were bred under the original environment as a control group. At the beginning of Phase II, we first dumped in the equal volumes of new soil bedding from the designated environments to the new sterile cages, then transferred the mice from their previous soil environments into the new cages. To avoid microbial contaminations cross cages and environments, each of mice were separately raised in one cage and all mice were maintained in three new-environments-dependent isolators from Day 60 to Day 90. Autoclaved and radiated (with Cobalt-60) diet and water fed to mice were strictly controlled during the whole experiment. The composition of the normal diet includes (g/kg): Casein: 140, L-Cystine:1.8, Corn Starch: 495.692, Maltodextrin: 125, Sucrose: 100, Cellulose: 50, Soybean Oil: 40, t-Butylhydroquinone: 0.008, Mineral Mix: 35, Vitamin Mix: 10, Choline Bitartrate: 2.5.

This study was approved and carried out in accordance with the guidelines for the care and use of laboratory animals by the Inner Mongolia Agricultural University of China. The study protocol was also approved by the Ethical Committee of Third Military Medical University (Chongqing China). Every effort was made to minimize animal suffering.

### Preparation of exogenous soil microbiota for mice cage bedding

The soil samples from the three different environments were packaged with aseptic bag while sampling, and then refrigerated at 4◦C before being transferred to the laboratory within 18 hours. Then the equal volumes of soil samples were transferred into each cage as initial bedding. Meanwhile, microbial DNA from the soil in each environment/group was extracted and sequenced (also see below). In both Phase I and II, the soil bedding in mice cages were replaced periodically (every 2 weeks) to remove the buildup of feces in the cages, respectively.

### Samples collection and metagenomic DNA extraction

For environmental (soil) samples, a total of 10 g of soil was mixed with 90 mL sterile NaCl solution (0.85%, w/v), and then were homogenized for 10 min into a homogenous suspension. A combination of PowerSoil DNA Isolation Kit (MO BIO, USA) and bead-beating method was used to extract DNA from the suspension. The integrity and purity of the extracted DNA were evaluated by 0.8% agarose gel electrophoresis and the OD 260/280 was determined with a Micro-Ultraviolet Spectrophotometer to determine its concentration. All extracted DNA samples were stored at −20°C. For fecal samples collected at Day 60 and 90 from each of mice, the QIAamp® DNA Stool Mini Kit (Qiagen, Hilden, Germany) was used for microbial DNA extraction. All of the DNA samples were stored at −80°C until further processing. In addition, stool samples were collected by stimulating anus directly without contact to the cage bedding.

### Data processing and taxonomy annotation

Samples were metagenomically sequenced as one library each multiplexed through Illumina HiSeq 2000 machines using the 2 × 100-bp paired-end read protocol. Clean data were trimmed and filtered by Trimmomatic^19^ using the parameter SLIDINGWINDOW: 30:20. Then, host (mouse) and diet (corn and soybean) DNA were then removed using Bowtie2^20^ to avoid their potential influence on the functional profiling. Taxonomic profiling was performed with Centrifuge v1.0.3^21^ (reference is based on NT database and the profiling was performed with default parameters) on the 186 metagenomic samples that passed the quality control. Centrifuge uses whole genomes for taxonomically profiling shotgun metagenomic data and to quantify the clades present in the microbiome with species-level resolution.

### Functional profiling and calculation of gene abundance

After discarding of host and diet DNA, clean reads were used as input for bowtie2 to align against the mice gut functional gene catalog,^22^ which includes annotation of all mice functional genes from Kyoto by Encyclopedia of Genes and Genomes (KEGG) databases. To estimate the relative gene abundance, we first normalized the sequencing coverage, the relative abundance instead of the raw read count was used to quantify the functional genes. The calculation process was as follows:

Step 1: Calculation of the copy number of each gene: b_i_ = x_i_/L_i_

Step 2: Calculation of the relative abundance of gene i: a_i_ = b_i_/∑_i_b_i_

a_i_: the relative abundance of gene i

b_i_: the copy number of gene i from sample N.

L_i_: the length of gene i.

x_i_: the number of mapped reads.

The calculations as mentioned above were completed using custom Python scripts.

### Statistical analysis

Statistical analyses were performed mainly using R and Python. Kruskal-Wallis test, Wilcoxon rank sum test and PERMANOVA test were performed in R with the standard kruskal.test, wilcox.test functions and the adonis function in the R package vegan, respectively. Rarefaction analysis and Shannon diversity index were used to estimate the richness and diversity of species. The relative abundances of differential taxonomic groups were visualized by “pheatmap” in the “pheatmap” R package. Differences in the relative abundances of taxonomic groups at the species level between samples were evaluated with Wilcoxon rank sum test. False discovery rate (FDR) values were estimated using the Benjamini-Hochberg method to control for multiple testing. *P*-values less than 0.05 were considered statistically significant. Differentially abundant functional genes were tested with DESeq2.^23^ To evaluate the enrichment of specific KEGG pathways, we calculated a reporter Z score (*Z*_adjustedpathway_) for each KEGG pathway based on the Z-scores of individual KOs involved. A reporter Z score of ≥2 (95% confidence according to normal distribution) was set as the detection threshold for a significantly enriched pathway.^24^ Whether the pathway modules were enriched in the desert, steppe or forest group was further determined by comparing the number of individual KOs that was enriched in the specific direction using ternary analysis (triple graph).

## Supplementary Material

Supplemental Material

## Data Availability

Raw sequencing reads for all 186 samples described in this project have been deposited in the NCBI Sequence Read Archive under accession no. PRJNA542998. Further information and requests for resources and reagents should be directed to and will be fulfilled by the corresponding author, H.Z. (hepingdd@vip.sina.com).

## References

[1] Lax S, Smith DP, Hampton-Marcell J, Owens SM, Handley KM, Scott NM, Gibbons SM, Larsen P, Shogan BD, Weiss S, et al. Longitudinal analysis of microbial interaction between humans and the indoor environment. Science. 2014;345(6200):1048–13. doi:10.1126/science.1254529. 25170151 PMC4337996

[2] Tasnim N, Abulizi N, Pither J, Hart MM, Gibson DL. Linking the Gut microbial ecosystem with the environment: does Gut health depend on where we live? Front Microbiol. 2017;8:1935. doi:10.3389/fmicb.2017.01935 PMC5635058 29056933

[3] Buffie CG, Pamer EG. Microbiota-mediated colonization resistance against intestinal pathogens. Nat Rev Immunol. 2013;13(11):790–801. doi:10.1038/nri3535. 24096337 PMC4194195

[4] Vo N, Tsai TC, Maxwell C, Carbonero F. Early exposure to agricultural soil accelerates the maturation of the early-life pig gut microbiota. Anaerobe. 2017;45:31–39. doi:10.1016/j.anaerobe.2017.02.022. 28249795

[5] Stein MM, Hrusch CL, Gozdz J, Igartua C, Pivniouk V, Murray SE, Ledford JG, Marques Dos Santos M, Anderson RL, Metwali N, et al. Innate immunity and asthma risk in amish and hutterite farm children. N Engl J Med. 2016;375(5):411–421. doi:10.1056/NEJMoa1508749. 27518660 PMC5137793

[6] Ottman N, Ruokolainen L, Suomalainen A, Sinkko H, Karisola P, Lehtimaki J, Lehto M, Hanski I, Alenius H, FyhrquistN. Soil exposure modifies the gut microbiota and supports immune tolerance in a mouse model. J Allergy Clin Immunol. 2019;143(3):1198–1206. doi:10.1016/j.jaci.2018.06.024. 30097187

[7] Gensollen T, Iyer SS, Kasper DL, Blumberg RS. How colonization by microbiota in early life shapes the immune system. Science. 2016;352(6285):539–544. doi:10.1126/science.aad9378. 27126036 PMC5050524

[8] Dey N, Wagner VE, Blanton LV, Cheng J, Fontana L, Haque R, Ahmed T, Gordon J. Regulators of gut motility revealed by a gnotobiotic model of diet-microbiome interactions related to travel. Cell. 2015;163(1):95–107. doi:10.1016/j.cell.2015.08.059. 26406373 PMC4583712

[9] Seedorf H, Griffin NW, Ridaura VK, Reyes A, Cheng J, Rey FE, Smith M, Simon G, Scheffrahn R, Woebken D. Bacteria from diverse habitats colonize and compete in the mouse gut. Cell. 2014;159(2):253–266. doi:10.1016/j.cell.2014.09.008. 25284151 PMC4194163

[10] Zhou W, Chow KH, Fleming E, Oh J. Selective colonization ability of human fecal microbes in different mouse gutenvironments. ISME J. 2019;13(3):805–823. doi:10.1038/s41396-018-0312-9. PMC6461746 30442907

[11] Brown EM, Wlodarska M, Willing BP, Vonaesch P, Han J, Reynolds LA, Arrieta M-C, Uhrig M, Scholz R, Partida O. Diet and specific microbial exposure trigger features of environmental enteropathy in a novel murine model. Nat Commun. 2015;6(1):7806. doi:10.1038/ncomms8806. 26241678 PMC4532793

[12] Dominguez-Bello MG, Godoy-Vitorino F, Knight R, Blaser MJ. Role of the microbiome in human development. Gut. 2019;68(6):1108–1114. doi:10.1136/gutjnl-2018-317503. 30670574 PMC6580755

[13] Sprockett D, Fukami T, Relman DA. Role of priority effects in the early-life assembly of the gut microbiota. Nat Rev Gastroenterol Hepatol. 2018;15(4):197–205. doi:10.1038/nrgastro.2017.173. 29362469 PMC6813786

[14] Miller ET, Svanback R, Bohannan BJM. Microbiomes as metacommunities: understanding host-associated microbes through metacommunity ecology. Trends Ecol Evol. 2018;33(12):926–935. doi:10.1016/j.tree.2018.09.002. 30266244

[15] Grieneisen LE, Charpentier MJE, Alberts SC, Blekhman R, Bradburd G, Tung J, Archie EA. Genes, geology and germs: gut microbiota across a primate hybrid zone are explained by site soil properties, not host species. Proc Biol Sci. 2019;286:20190431. 31014219 10.1098/rspb.2019.0431 PMC6501927

[16] Knight R, Vrbanac A, Taylor BC, Aksenov A, Callewaert C, Debelius J, Gonzalez A, Kosciolek T, McCall L-I, McDonald D. Best practices for analysing microbiomes. Nat Rev Microbiol. 2018;16(7):410–422. doi:10.1038/s41579-018-0029-9. 29795328

[17] Integrative HMPRNC. The integrative human microbiome project. Nature. 2019;569(7758):641–648. doi:10.1038/s41586-019-1238-8. 31142853 PMC6784865

[18] Martinez I, Maldonado-Gomez MX, Gomes-Neto JC, Kittana H, Ding H, Schmaltz R, Joglekar P, Cardona RJ, Marstelle NL, Kembel SW, et al. Experimental evaluation of the importance of colonization history in early-life gut microbiota assembly. Elife. 2018;7:e36521. doi:10.7554/eLife.36521. PMC6143339 30226190

[19] Bolger AM, Lohse M, Usadel B. Trimmomatic: a flexible trimmer for Illumina sequence data. Bioinformatics. 2014;30(15):2114–2120. doi:10.1093/bioinformatics/btu170. 24695404 PMC4103590

[20] Langmead B, Wilks C, Antonescu V, Charles R, Hancock J. Scaling read aligners to hundreds of threads on general-purpose processors. Bioinformatics. 2019;35(3):421–432. doi:10.1093/bioinformatics/bty648. 30020410 PMC6361242

[21] Kim D, Song L, Breitwieser FP, Salzberg SL. Centrifuge: rapid and sensitive classification of metagenomic sequences. Genome Res. 2016;26(12):1721–1729. doi:10.1101/gr.210641.116. 27852649 PMC5131823

[22] Xiao L, Feng Q, Liang SS, Sonne SB, Xia ZK, Qiu XM, Li X, Long H, Zhang J, Zhang D, et al. A catalog of the mouse gut metagenome. Nat Biotechnol. 2015;33(10):1103-+. doi:10.1038/nbt.3353. 26414350

[23] Love MI, Huber W, Anders S. Moderated estimation of fold change and dispersion for RNA-seq data with DESeq2. Genome Biol. 2014;15(12):550. doi:10.1186/s13059-014-0550-8. PMC4302049 25516281

[24] Feng Q, Liang SS, Jia HJ, Stadlmayr A, Tang LQ, Lan Z, Zhang D, Xia HH, Xu XY, Jie ZY, et al. Gut microbiome development along the colorectal adenoma-carcinoma sequence. Nat Commun. 2015;6:6528. doi:10.1038/ncomms7528. 25758642

