# Supplementary material for: Exposure to soil environments during earlier life stages is distinguishable in the gut microbiome of adult mice
Source: Gut Microbes. 2020 Dec 31;13(1):1830699. doi: 10.1080/19490976.2020.1830699 (PMC7781656; doi:10.1080/19490976.2020.1830699)
Supplement: Supplemental Material [file KGMI_A_1830699_SM1615.zip › Supplementary information/Supplemental Information titles and legends.docx]

**Supplemental Information titles and legends**

**Figure S1. Comparison of differential species among different environments at Day 60 and Day 90 grouped by post-transferring soil environments (a) or birthplace (b).**

**Figure S2. Significantly enriched pathways among three groups at Day 60.** Grey points refer to those KOs who were not significantly changed among three groups and red for those significantly changed KOs. The closer the point to the apex of triangle the higher abundance of this KO in corresponding group.

**Figure S3. Significantly enriched pathways among current environments at Day 90.**

**Figure S4. Significantly enriched pathways among birthplaces at Day 90.**

Figure S5. Source tracking analysis did not support any contamination in the gut microbiotas of mice at both Day 60 and Day 90.
