# Supplementary figures and images for: Exposure to soil environments during earlier life stages is distinguishable in the gut microbiome of adult mice
Source: Gut Microbes. 2020 Dec 31;13(1):1830699. doi: 10.1080/19490976.2020.1830699 (PMC7781656; doi:10.1080/19490976.2020.1830699)

Figure S1

a

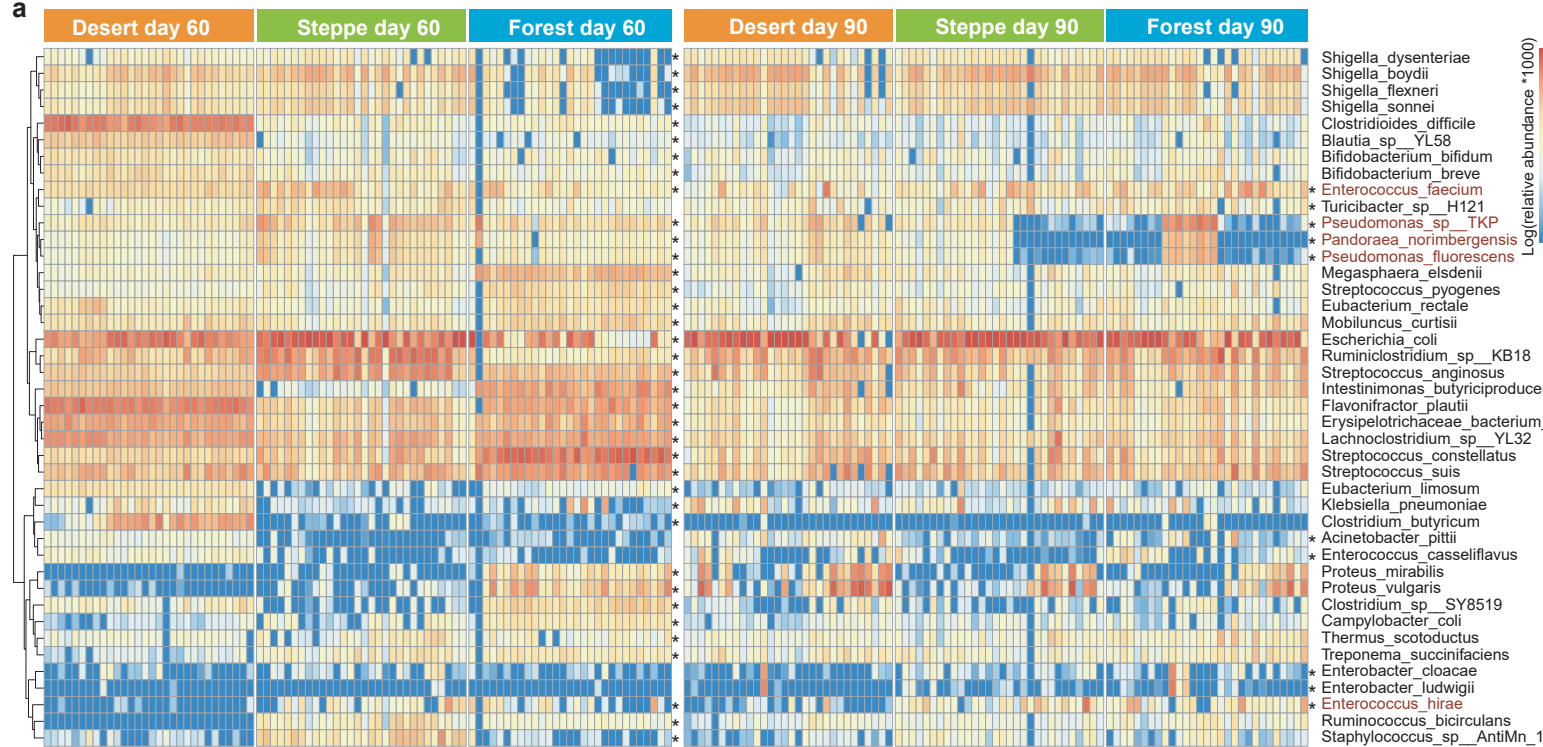

b

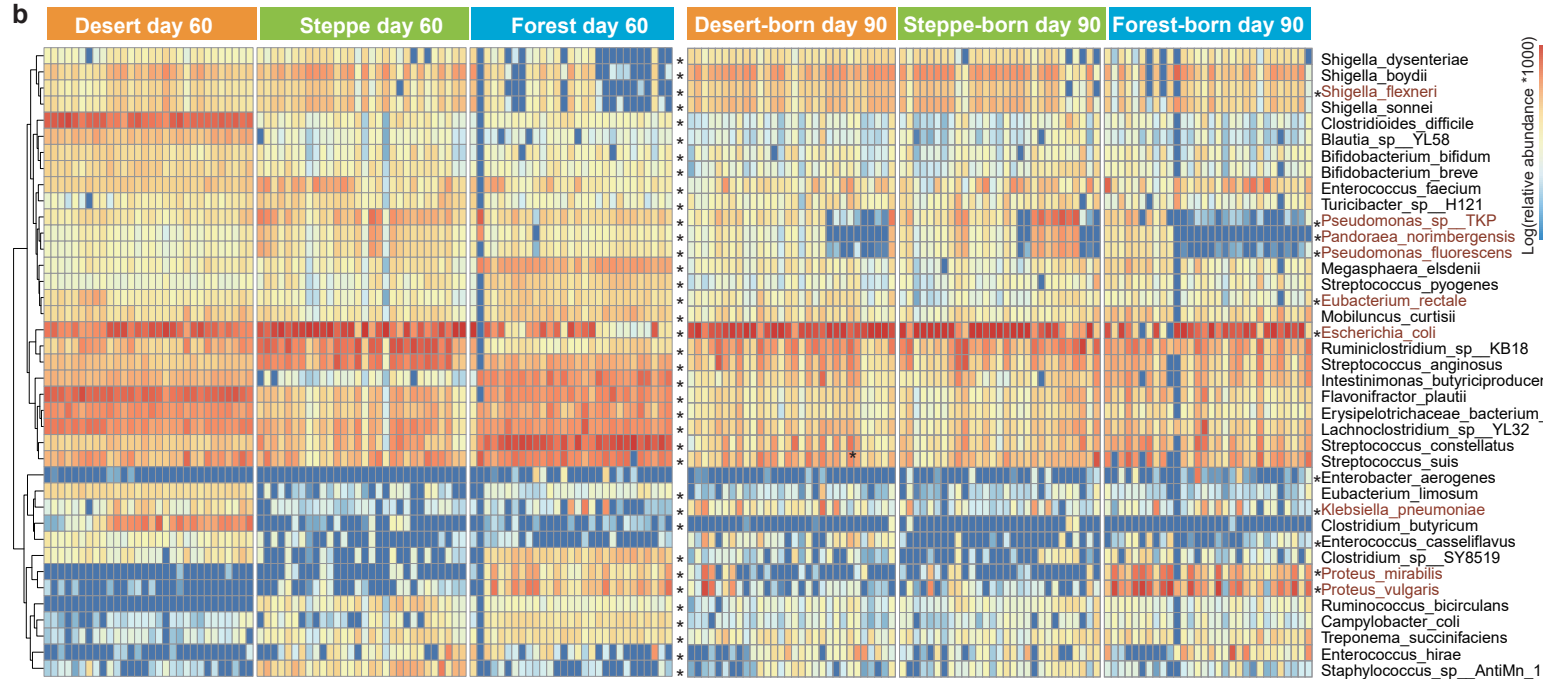

Supplement: Supplemental Material [file KGMI_A_1830699_SM1615.zip › Supplementary information/FigureS1.pdf]

Figuyre S2

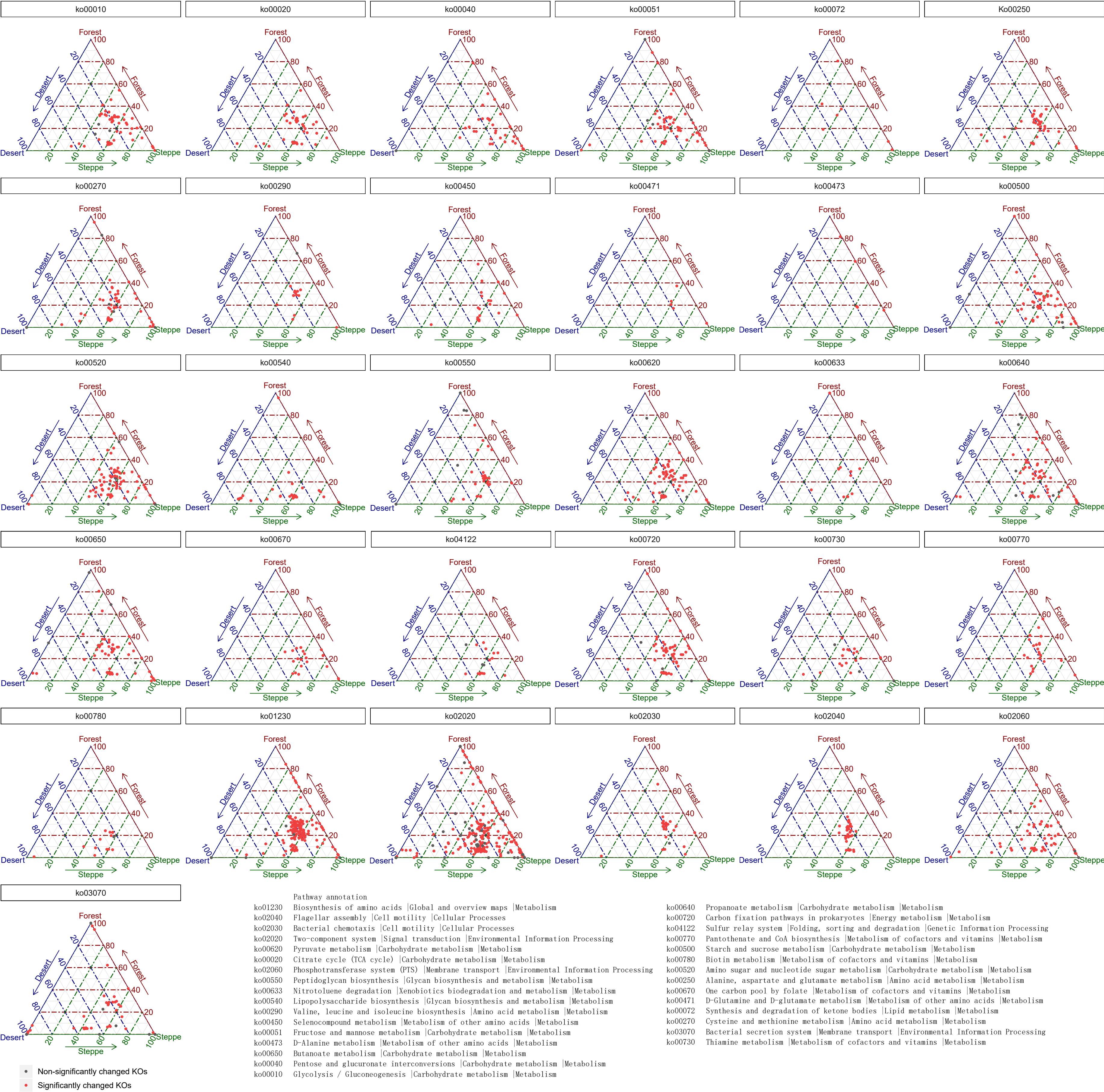

Supplement: Supplemental Material [file KGMI_A_1830699_SM1615.zip › Supplementary information/FigureS2.pdf]

Figure S3

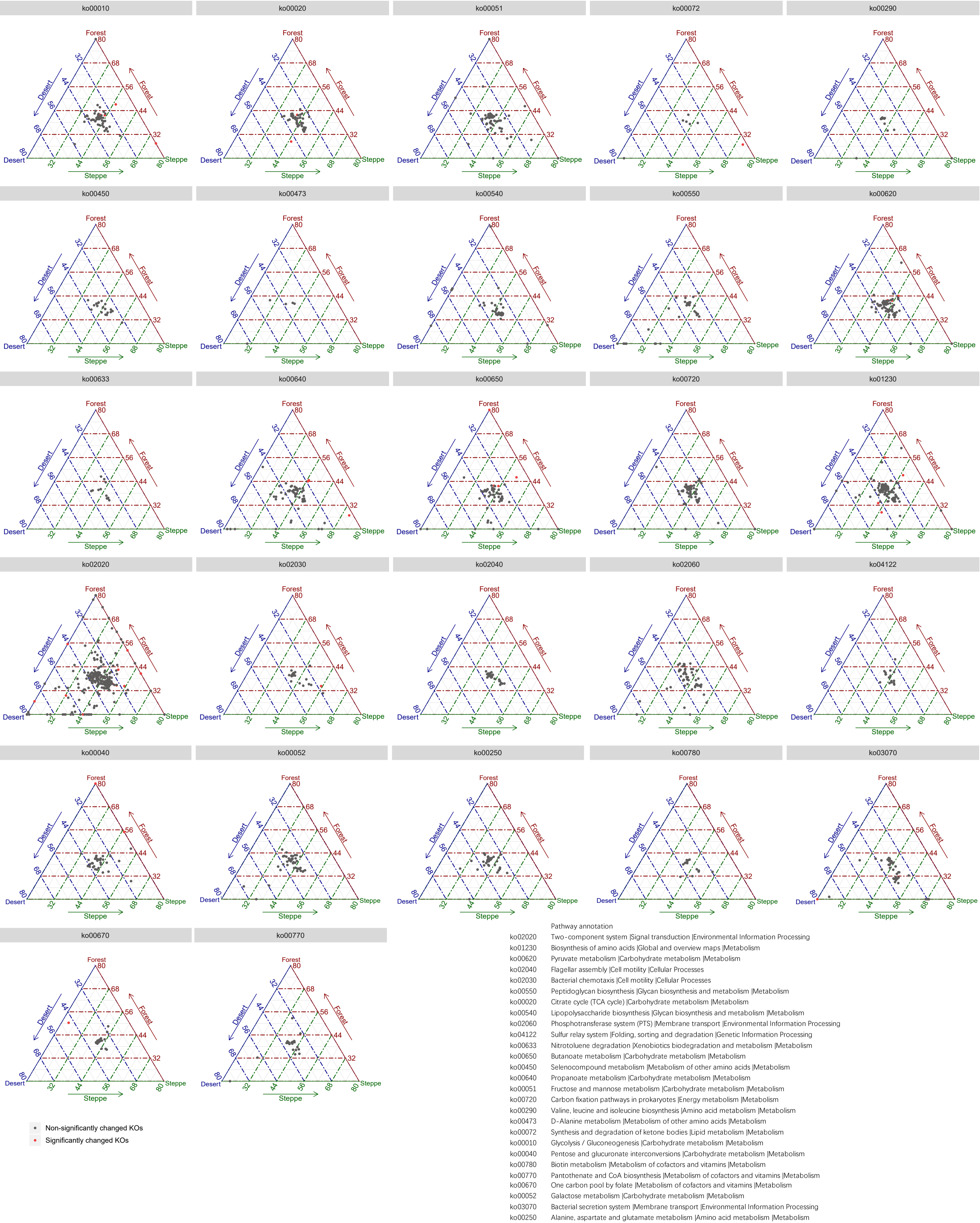

Supplement: Supplemental Material [file KGMI_A_1830699_SM1615.zip › Supplementary information/FigureS3.pdf]

Figure S4

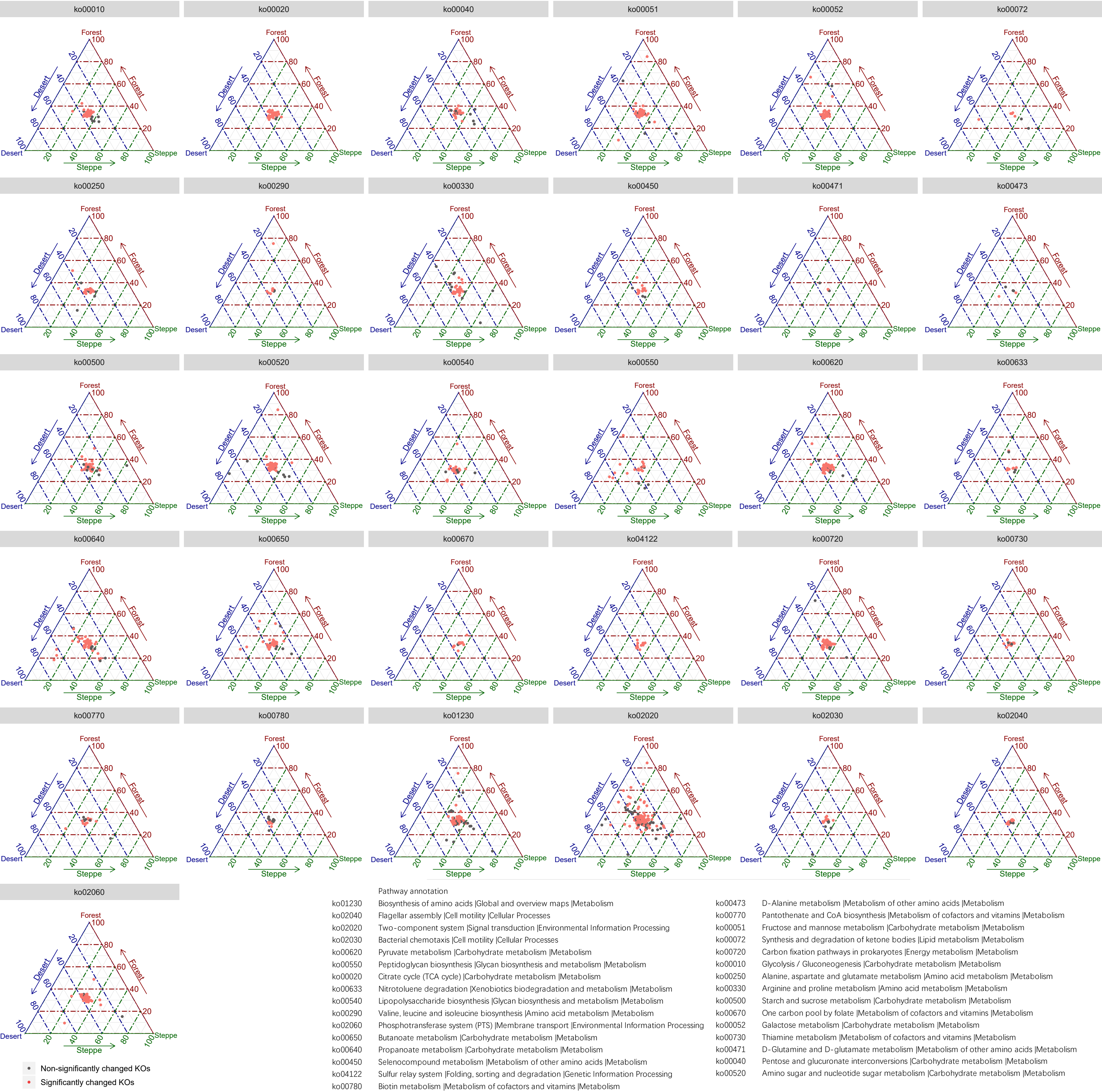

• Non-significantly changed KOs

• Significantly changed KOs

Supplement: Supplemental Material [file KGMI_A_1830699_SM1615.zip › Supplementary information/FigureS4.pdf]

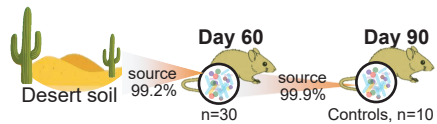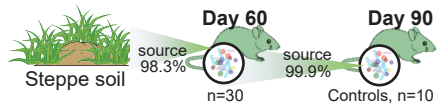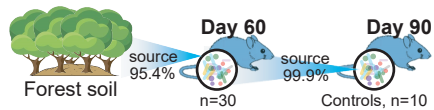

Supplement: Supplemental Material [file KGMI_A_1830699_SM1615.zip › Supplementary information/FigureS5.pdf]
